# Changes in Inpatient and Day Patient Treatment during the COVID-19 Pandemic: Insights and Outlook Results of a Study in an Adult Psychiatry Clinic in Germany

**DOI:** 10.1192/j.eurpsy.2022.1263

**Published:** 2022-09-01

**Authors:** T. Barth, B. Voigtländer

**Affiliations:** 1 Klinikum Chemnitz, Psychiatry, Chemnitz, Germany; 2 Klinikum Chemnitz, Psychiatry, Dresden, Germany

**Keywords:** covid, Changes in Pandemic Situation, Clinical Treatment

## Abstract

**Introduction:**

Possible effects of pandemic-related restrictions and adjustments in psychiatric treatment are currently the focus of interest. This article addresses developments in this respect in our clinic.

**Objectives:**

Changes in clinical practice that occurred during the COVID-19 pandemic are to be analysed with regard to possible risks for affected patients.

**Methods:**

A clinic-internal analysis was carried out, focusing on comparing a period during the COVID-19 pandemic (01.11.2020-30.04.2021) and a pre-pandemic reference period (01.11.2018-30.04.2019).

**Results:**

Following trends were observed during the pandemic period: a. Day patient treatment: - The treatment volume fell to 44%. Notable reductions in the number of treatment cases with main diagnoses [ICD-10] F10.- and F30-F39 by >65% were measured. b. Inpatient treatment: - no significant changes regarding socio-demographic patient data and concerning the type of admission and discharge, - detection of coronavirus SARS-CoV-2 by PCR test in 4.7% of the cases, - a decline in the treatment volume to 87% due to 8% decrease in the number of cases and 5% decrease in the ALOS, with patients with the main diagnosis [ICD-10] F10.- were most affected, - increases regarding the percentage of cases with the main diagnosis [ICD-10] F40-F48 (p<0.05) and in the ALOS of this patient group (by 31%), - a significant rise (p< 0.01) in the ratio of restrained treatment cases.

**Conclusions:**

Related to the decrease in treatment volume, the increase in psychiatric intensive treatment and possible risks in terms of the care situation for patients with the main diagnoses [ICD-10] F10.-, F30-F39 and F40-F48 should be discussed in particular.

**Disclosure:**

No significant relationships.

